# Model Organisms: Grandeur in the Diversity of Aging Organisms

**DOI:** 10.1093/geroni/igaa057.2667

**Published:** 2020-12-16

**Authors:** Anne Brunet

## Abstract

Aging is a complex process that converts vigorous and healthy individuals into frail and decrepit ones, with increased susceptibility to a constellation of diseases. Human aging is influenced by many factors, including genetics, environment, lifestyle, sex, and socio-economic status. While aspects of aging can be studied directly in humans, discovering the causative factors that modulate this process often requires interventions and modeling. Traditional models will likely continue to provide a wealth of translatable information. Studying ‘extremophiles’ has exciting potential for providing new concepts that could be implemented for lifespan regulation. The development of new experimental models uniquely tailored to aging studies is also an essential step. This symposium will discuss African killifish, planarian, naked mole rats, and domestic dogs as new models for aging and exceptional longevity and rejuvenation. The iteration between new models and humans could be particularly helpful in delineating strategies to promote healthy aging and extend the disease-free portion of life.

